# Exploring adaptive capacity to arid heat in remote First Nations communities in Central Australia

**DOI:** 10.1038/s41598-026-40677-2

**Published:** 2026-02-21

**Authors:** Manoj Bhatta, Gloria Baliva, Sophie Pascoe, Mohammad Radwanur Talukder, Vahab Baghbanian, Deborah Russell, Linda Ford, Alan Cass, John Wakerman, Supriya Mathew

**Affiliations:** 1 Menzies School of Health Research, Charles Darwin University, Alice Springs, NT Australia; 2 Menzies School of Health Research, Charles Darwin University, Darwin, NT Australia; 3 Leukaemia Foundation, Adelaide, SA Australia; 4 Central Australian Aboriginal Congress, Alice Springs, NT Australia; 5 Northern Institute, Charles Darwin University, Darwin, NT Australia

**Keywords:** Climate adaptation, Vulnerability, Resilience, Indigenous people, Heat, Climate sciences, Developing world, Environmental social sciences, Environmental studies, Geography, Geography

## Abstract

Extreme heat is associated with increased mortality and high health service demands. Despite a substantial body of heat-health research in Australia, there is limited evidence on hot weather-specific impacts on First Nations people living in remote communities. Here we used one-on-one yarning sessions, a First Nations form of knowledge sharing, with 30 participants (*n* = 30) to explore factors influencing vulnerability and resilience to hot weather in four remote First Nations communities of Central Australia. Using purposive and snowball sampling, participants were recruited to document lived experiences of extreme heat and related adaptive practices. A deductive coding approach aligned with a vulnerability-resilience framework was applied to the data. Participants described how hot weather affected their physical and mental health, daily activities, sleep, and cultural practices. Participants highlighted increased risks associated with hot weather for children, older people, women, and people with pre-existing medical conditions or disabilities. Although participants reported using a range of cooling strategies, including air conditioning, shifting activities to cooler parts of the day, resting under shade trees, and swimming in nearby waterholes, the effectiveness of these strategies was constrained by key vulnerabilities that increased heat-related health risks. These vulnerabilities were primarily linked to poor housing and energy insecurity that affected indoor thermal comfort, limited shaded outdoor areas or heat refuges, and certain cooling practices, such as consumption of sugar-containing cold beverages. Our findings indicate that investments in local infrastructure, reinforcement of adaptive knowledge, and co-produced knowledge strategies are essential for climate-resilient remote communities.

**Supplementary Information:**

The online version contains supplementary material available at 10.1038/s41598-026-40677-2.

## Introduction

Climate change has increased the frequency and intensity of heatwaves globally^1^. Currently, nearly 30% of the global population experiences extreme heat for at least 20 days annually, posing serious health risks^2^. For Australia, the number of record hot days has doubled over the last 50 years, which means, on average, there are almost 12 more days per year with a temperature over 35 °C^3^. Heatwaves have become almost five times more frequent since the 1950s^4^. Heatwaves have also accounted for about 55% of all disaster-related deaths in the past century, exceeding the combined toll of all other natural disasters^5^. The health risks associated with extreme hot weather range from mild health symptoms, such as heat rash and cramps, to more serious health issues, including exhaustion, dehydration, and heat stroke, which could result in death^6^. Young children, older people, outdoor workers, people living in rural and remote areas and people with pre-existing medical conditions have been observed to be most at risk of heat-related health effects^7^.

First Nations people living in remote locations of Australia are at substantially increased risk of heat-related health effects due to limited adaptation infrastructure^8^. Poor housing infrastructure, energy poverty and energy insecurity have been reported as key heat-related adaptation concerns in remote Australia^8^. Health and well-being disparities, influenced by colonisation and Western biomedical models^9^, further add to the health risks. In many remote inland communities, the annual number of hot days is already at par with temperature projections for 2090^10^, which makes it essential to invest in risk reduction measures. Despite the increasing climate-related health threats and the importance of First Nations’ knowledge about climate adaptation, there are limited primary studies on this topic^11^. The need to include First Nations perspectives and knowledge is gaining traction globally, with the Intergovernmental Panel on Climate Change Seventh Assessment Report (IPCC AR7) specifically highlighting the importance of including First Nations knowledge systems and experts^1^.

Vulnerability to extreme heat can be conceptualised through both infrastructural and social dimensions^12,13^. Infrastructural vulnerability reflects the material conditions that shape exposure and thermal comfort, including housing quality, energy access, cooling infrastructure, and the availability of shaded communal spaces. Social vulnerability reflects the social, economic, and institutional conditions that shape sensitivity and adaptive capacity, including health inequities, access to services, governance arrangements, and the ways knowledge, resources, and support circulate within households and community networks^14^. Distinguishing these dimensions is important because adaptation actions that prioritise infrastructure can reduce exposure, yet may do little to address underlying social drivers of risk or strengthen adaptive capacity if social and cultural determinants are not concurrently considered^15,16^. Against this backdrop, shifting from a deficit-focused vulnerability narrative to a strength-based resilience framing^17^ is important for effective climate adaptation in remote First Nations communities. Using a vulnerability–resilience lens, this study examines how heat-related risks in remote Central Australian communities arise through the interaction of infrastructural and social conditions, while also documenting locally grounded strengths, adaptive practices, and lived knowledge articulated by First Nations participants. In doing so, the study provides empirical insight into how adaptive capacity is experienced and enacted in arid, resource-constrained settings, addressing a key gap in the Australian heat–health literature.

## Methods

**Fig. 1:**
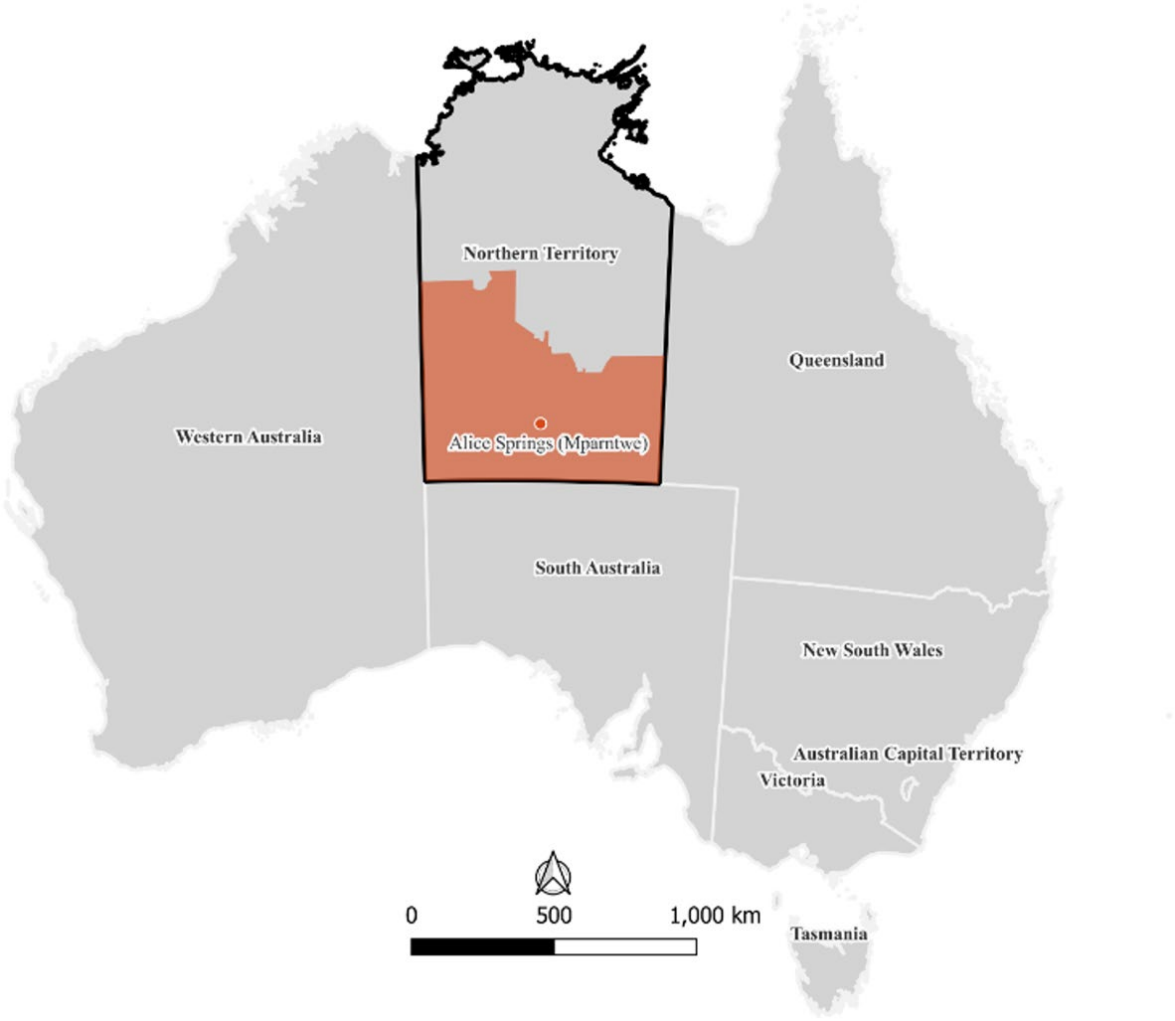
Location of the study area in Central Australia, Northern Territory, Australia. The study region is shown at a generalised spatial scale to protect confidentiality. State and territory boundaries and the location of Alice Springs (Mparntwe) are shown for geographic context.

This study was undertaken in Central Australia (Fig. 1), which has a desert climate. Over the last five years, the region has consistently recorded high numbers of extremely hot days, averaging 113 (92–137) days per year at 35 °C or above, and 33 (10–56) days reaching 40 °C or more^18^. The region covers approximately 551,218 km^2^ and borders three Australian jurisdictions. The region is home to nearly 40,000 people, of which 40% identify as First Nations^19^. The population is highly dispersed, with the four participating communities located between 21 and 240 km from the remote service town of Alice Springs (Mparntwe). Public transport is largely absent, and many communities face major barriers to accessing timely and appropriate health services^20^. These systemic challenges are layered over long-standing health inequalities, with the region experiencing a high burden of chronic disease and preventable health conditions^20^.

This study employed an ethnographic approach, with primary data collected and interpreted using multiple, complementary methods. The field research team documented observations of people’s daily activities, and locally available adaptation infrastructure across the study sites. These observations were conducted in public and communal spaces within each community, including outdoor gathering areas and shared facilities, with a focus on hot weather exposure and adaptation. Observations occurred naturally during field visits to each community, including on arrival and while waiting for cultural navigators, with observation periods varying by site. The field research team consisted of a Central Australian First Nations researcher (GB) with substantial research experience engaging with communities in Central Australia, and a non-Indigenous researcher (MB; long-term local resident researcher, > 3 years), enabling culturally informed engagement and reflexive interpretation throughout the study.

Primary qualitative data were collected through one-on-one yarning sessions with community residents to document local perceptions and lived experiences of hot weather. Yarning is a First Nations cultural practice and qualitative research method characterised by open, conversational dialogue that emphasises relationality, trust, and shared understanding rather than structured questioning^21,22^. As a research approach, yarning supports culturally safe knowledge sharing and is well suited to research conducted with First Nations communities.

A yarning circle (a collaborative discussion that prioritises First Nations ways of knowing and relationship building)^22^ was subsequently held with nine community participants as a culturally grounded process to support interpretation and contextualisation of the findings generated from the one-on-one yarning sessions across the four study sites. Participants included a subset of individuals who had taken part in the one-on-one yarning sessions, as well as additional community representatives, enabling collective reflection on preliminary findings. Unlike the one-on-one yarning sessions, the yarning circle was facilitated as a group discussion guided by emerging themes rather than a structured interview guide. This process did not involve the collection of new primary data but supported contextual accuracy and strengthened interpretive rigour.

In each community, participants were selected purposively, using snowball sampling techniques^23^, with assistance from First Nations cultural navigators (local male and female community members).This approach was used to recruit community members with lived experience of hot weather impacts and adaptation practices in remote settings, with attention to including residents across different age groups and genders. Inclusion criteria comprised adults (≥ 18 years) residing in the participating communities who were willing to share experiences related to hot weather and daily life, while exclusion criteria were minimal and limited to individuals unable to provide informed consent or those who did not wish to participate.

First Nations cultural navigators supported culturally appropriate recruitment processes and checked the cultural appropriateness of research protocols and yarning guides, consistent with best practices for research involving Traditional Ecological Knowledge (TEK) and Indigenous communities^24,25^. One-on-one yarning sessions were led by a local First Nations researcher (GB) to support culturally safe engagement and minimise potential power dynamics during data collection. One-on-one yarning sessions also supported participant-led discussion and helped manage intra-community power dynamics and cultural avoidance considerations. First Nations cultural navigators translated interview processes and questions into the local language when required by participants.

The data collected were audio-recorded (with consent) and supplemented by field notes to capture non-verbal cues and other contextual information observed by the research team. As a token of appreciation for their time and contribution, participants received a voucher equivalent to $50.

The study received human research ethical clearance from the Northern Territory Human Research Ethics Committee (NT HREC 2022–4473). All methods were performed in accordance with the relevant guidelines and regulations, including the NHMRC National Statement on Ethical Conduct in Human Research and the AIATSIS Code of Ethics for Aboriginal and Torres Strait Islander Research. During the informed consent process, participants were advised that they could choose not to share any TEK. Where such knowledge was shared, the research team clarified that intellectual ownership remained with the community. Participants provided consent for the use of de-identified information for research purposes only.

The vulnerability–resilience framework was selected to guide this study because it enables examination of both heat-related vulnerabilities and existing adaptive strengths within communities. The framework, as conceptualised by^26^, integrates narratives of vulnerability and resilience for remote and marginalised social–ecological systems and was developed to broaden adaptation thinking beyond deficit-based approaches. Its emphasis on exposure, sensitivity, adaptive capacity, and learning processes is well suited to the climatic, social, and infrastructural conditions that characterise of remote Central Australian communities. The framework supports analysis of everyday coping practices, social relations, and place-based practices that contribute to resilience in complex social–ecological contexts.

The application of the framework in this study was discussed with the local First Nations researcher (GB) to ensure cultural appropriateness and alignment with Indigenous ways of understanding vulnerability, resilience, and adaptation. Alternative approaches that focus primarily on biophysical exposure, hazard risk, or infrastructural vulnerability were considered but deemed less suitable, as they tend to underrepresent social processes, cultural practices, and locally grounded adaptive knowledge central to this study^12,13^. While the vulnerability–resilience framework is primarily descriptive and does not explicitly assess long-term adaptation practices, it provides a flexible and integrative structure well aligned with qualitative, ethnographic, and Indigenous-led research approaches focused on lived experience and everyday adaptive responses. These limitations are considered in the interpretation of findings.

The audio-recordings were transcribed verbatim and analysed using a deductive approach^27^, with codes and themes organised in accordance with the vulnerability-resilience framework^26^. Accordingly, the analytic categories and associated subcategories used in the Results were guided by this framework and the one-to-one yarning guide, rather than emerging inductively during analysis. The one-to-one yarning guide (Supplementary File S1) and deductive codebook (Supplementary Table S1) are provided in the Supplementary Materials. GB, who led the data collection, undertook transcription and primary coding through close engagement with the data, including repeated listening to audio-recordings and use of interview notes to support manual coding. This approach facilitated contextual familiarity and reflexive interpretation of participants’ accounts.

NVivo software (NVivo 14) was used to support systematic organisation of the coded data and to facilitate co-coding. A subset of transcripts (10%) was independently coded by SM using NVivo, and discrepancies were discussed collaboratively with GB to reach consensus. Given the qualitative and interpretive nature of the analysis, formal quantitative intercoder reliability metrics were not calculated; instead, reflexive discussion and consensus were used to enhance analytic consistency and credibility. An additional layer of interpretive support was provided through the yarning circle with community representatives and cultural navigators, which helped refine and contextualise key themes and supported respectful representation of participants’ perspectives.

## Results

### Non-local researchers’ observations across study sites

Observations were conducted across all four study sites. The observations are presented as contextual insights rather than uniform patterns. Some common features were observed across communities, including limited shaded public spaces, uneven vegetation around housing, and reduced outdoor activity during periods of extreme heat. However, there was clear variation within and between communities in the extent and form of these features and in daily activities.

The data collection was conducted between December 2023 and May 2024, which included some of the hottest months of the calendar year 2023/24 in Central Australia. Many days during fieldwork recorded maximum temperatures of more than 40 °C (104 °F). The non-local research team (*n* = 2) noted heat fatigue while getting in and out of air-conditioned cars.

Some residents had placed bed frames outside their houses. Some elderly people were observed sitting outside under the shade of trees during the day. Many households had shade cloths on their front porch or verandas. Some houses had above-ground pools (non-permanent structures) in the yard, often without fencing. Transitional housing was present in several communities to accommodate residents during housing maintenance or upgrades. These dwellings generally lacked shaded outdoor areas, although they were equipped with solar hot water systems and painted white roofs. In contrast, older houses more often had larger covered verandas and mature trees around yards.

Solar hot water systems were commonly observed on residential and public buildings across communities. However, playgrounds often lacked adequate shade. Some small shelters with seating provided shade for part of the day, but drinking taps were rarely observed near sporting grounds, and public water fountains were not observed in several communities. Communities lacked treelined or shaded footpaths. A significant presence of buffel grass, an introduced species in arid Australia, was observed in and around the communities.

In each community, the general store appeared to function as a main meeting place for residents. Beyond this, there was limited movement of people observed in community spaces during research visits. Council workers were observed undertaking outdoor maintenance activities during the hottest part of the day, all wearing hats and protective clothing.

### Community one-to-one yarns

Thirty First Nations participants [Site 1 (*n* = 9); Site 2 (*n* = 8); Site 3 (*n* = 7); Site 4 (*n* = 6)] from four communities participated in individual yarns. The majority were female respondents (70%). The participants ranged from 24 to 74 years, with 60% participants aged 49 or older. The community responses were coded against: (i) heat-related vulnerabilities; (ii) resilience to hot weather; and (iii) community recommendations on improving adaptive capacity. Selective illustrative participant quotations supporting each theme and sub-theme are included in the main text, with additional quotations provided in Supplementary Table S2.

1. Heat-related vulnerabilities

Participants linked heat-related vulnerabilities to people’s health and well-being, constraints related to infrastructure and services, environmental changes, and broader socio-economic conditions, including access to heat-health information.

- Health and well-being

Participants in all four sites noted the pronounced change in weather patterns, expressing their concerns about the weather getting increasingly hot. Hot weather was frequently described as negatively affecting the health of community members. Children, older people, pregnant women, disabled members of the community and people with pre-existing medical conditions were considered at higher risk.

“*Older people and kids get irritated and angry when it’s too hot*” [Site 2.3].

*“My daughter has asthma*,* she feels out of breath when she is in the heat”* [Site 3.3].

Nausea, headache, dehydration, difficulty breathing, vomiting, nosebleeds, heatstroke, tiredness, sunburn, agitation, and foot burn were the key words used to describe heat-related health symptoms. Participants were worried about the community’s well-being as hot weather affected people’s involvement in outdoor activities, including travel to meet family and involvement in various cultural practices.

“*It really impacts me*,* you know*,* because you can’t go out hunting*,* it’s just too hot. And*,* yeah*,* in the summer*,* can’t go out anywhere or go*,* visit families*,* it’s too hot*,* so probably in the wintertime*,* it’s good to go”* [Site 4.1].

(b) Infrastructure to adapt

Participants across all sites highlighted that their communities did not have the necessary infrastructure to support adaptation to heat. Socio-economic marginalisation was perceived to increase heat-related vulnerabilities. Frequent power disconnections caused by the inability to purchase pre-paid residential power cards resulted in challenges to storing food in refrigerators. This impacted health and further stretched household budgets.

*“Sometimes*,* when they [food] look good*,* we eat; if they get no good*,* we end up chucking it in the bin”* [Site 1.5].

Household-level cooling was affected by overcrowding, damaged fixtures or malfunctioning appliances, untimely installation and repair of air conditioners, power outage issues, and power and appliance affordability concerns.

*“Sometimes no money*,* no power*,* no money*,* we sleep outside”* [Site 2.1].

*“Cooks her breakfast*,* lunch and dinner outside*,* in the heat*,* because the stove is not working*,* she’s been waiting to get her stove fixed for a long time.”* [Site 2.5, translated by cultural navigator].

There were mixed responses about outdoor cooling spaces/structures within communities. Some participants stated that while there was shade structures built in the community, they were not easily accessible during hot weather or were unevenly distributed.

“*I got a tree only outside the fence*,* not in my yard”* [Site 3.1].

Accessing the local health clinic during hot weather periods was found to be challenging, even if it meant walking for short distances. For some communities, clinics were only open two days a week, which meant appointments would have to be made during specified days.

*“The clinic only opens two days but should open five days a week. The ambulance can pick people up from their homes and take them to the clinic on hot days. If they walk*,* they get dizzy”* [Site 2.6].

(c) Environmental changes

Elderly participants described observing changes in local environmental and weather patterns. Increased evaporation rates, reduced rainfall, declining water availability, and decreased availability of bush tucker (traditional native foods derived from local plant and animal species), increasingly displaced by dense growth of buffel grass, were described as affecting people’s ability to cool down using natural sources (e.g., swimming in nearby waterholes).

*“There used to be waterholes that people maintained*,* waterholes always got water in them. Now it’s dry”* [Site 2.2].

(d) Knowledge and awareness of heat-related health risks

Participants described variation in heat health awareness within communities and suggested this may influence how people recognise and respond to heat-related symptoms. Participants also described limited heat-health promotion as potentially contributing to cooling practices that are less effective during hot weather. Some participants mentioned that in hot weather, some community members often drink cold alcoholic beverages to cool down.

*“The only thing that’s missing out is the people in the community who are not really into literacy and numeracy*,* who don’t know all this. Where you still need the health department to come and do promotions on those sorts of issues now*,* like blood sugar levels*,* diabetes*,* and hypertension”* [Site 1.1].

*“They [some community members] drink alcohol outside the community and come back in*,* want to fight and argue and get stressed out from heat”* [Site 3.4].

Participants highlighted that diabetes was prevalent in remote communities, and the practice of consuming soft drinks to cool down during hot weather was a key risk factor to health.

*“Everyone loves Coke; we drink it a lot during the summer when it’s hot*” [Site 2.7].

2. Resilience to hot weather

Participants described a range of strengths and adaptive responses that supported resilience to hot weather, including the role of primary health care (PHC) services, household and community-level cooling practices, public infrastructure and social support systems, and access to weather and heat-health information.

- Strong primary care

Participants perceived that clinic use increased during hot weather, and clinics were still providing effective services to the communities. In some sites, the clinic staff ensured client transportation services were provided. Some clinics were also responsible for providing health warnings during heatwaves.

*“Yeah*,* they always go picking up”* [Site 4.1].

*“We get information from the clinic (verbally) about weather warnings*,* like if it’s going to be a heatwave in the next couple of days and stay out of the heat and that”* [Site 3.1].

(b) Adaptation practices adopted by residents

Participants across the communities adopted diverse indoor cooling strategies, including using air conditioners and electric fans, showering regularly, opening windows, using makeshift curtains to block the sun and keep out the heat, temporary shade structures, and mopping the house floors. Outdoor cooling strategies included sleeping outside at night, sitting under the shade of trees, hosing the yard, and swimming in water holes around the communities and in the community pools. Some community participants described how:

*“When the water levels are really low*,* we dig a soakage hole in the creek bed to have a soakage”* [Site 2.2].

*“They (community members) sit down outside in the shade*,* they got their own built-in shade or bower shelters that someone built*,* so most of our people all the time sit down outside when no power”* [Site 1.1].

Participants commonly described shifting outdoor activities to cooler periods of the day. These included changing clinic visit times and shifting physically intensive activities to either early in the morning or later in the evening.

*“Older people visit the clinic only in the morning during hot weather”* [Site 2.4].

*“When it is really hot*,* we go hunting early in the morning. You know once you kill the animal*,* you sit down under a tree relaxing till about lunchtime”* [Site 3.6].

Many participants described drinking plenty of water (and iced water) to cool down during hot weather. Consumption of hot tea was also used as an evaporative cooling strategy. Locally available bush tucker, for example, Witchetty grub (moth larvae), honeypot ants, bush bananas (*Elangwa*), sugar bag (bush honey), bush berries, and bush orange (*Whagi*), were also consumed to cool down.

*“Yeah*,* I drink hot tea*,* that will make me sweat*,* that will make me cool when the air-con is on or wind is blown on you”* [Site 2.2].

*Yeah*,* people collect honey ants and Witchetty grub during summer”* [Site 3.1].

(c) Public infrastructure and local support systems

Participants stated that schools, clinics, aged care centers, art centers and community stores have better facilities to cool down than houses, and that children and elderly people regularly used these spaces during hot weather.

*“Old people centers (aged care centers) provide meals. They do their washing*,* blankets*,* and clothes. They can have a shower there too”* [Site 1.6].

*“When it is hot*,* I go to the shop and stay in the shop for a bit longer so I can cool down”* [Site 2.8].

There was a sense of social connectedness prevalent among community members, which meant power cards and food were shared during times of hardship. For example, some people may use a neighbour’s fridge to store food and drink during a power disconnection.

*“If we have meat*,* we will take it to someone else’s house and store it for the night or till whenever power comes back on”* [Site 2.1].

(d) Heat-health communication

People in the study communities received weather-related information from various television channels (ABC TV and Indigenous Community Television (IC TV), Imparja TV), through the radio (Central Australian Aboriginal Media Association (CAAMA) radio station) and local services (e.g. council and clinic). Some elderly participants stressed that they still followed the traditional ways of predicting hot weather.

*“A big bunch of stars (seven sisters) altogether in the sky*,* if it’s northeast in the sky*,* that means it’s going to be long hot summer. But if it’s northwest in the sky it’s going to be not so hot it’s going to be cooler. The summer is going to be cooler”* [Site 2.6].

*“Sometimes the weather information is provided by the shire [Council] mob”* [Site 3.4].

3. Community recommendations on improving adaptive capacity

Participants identified a range of community-led recommendations to strengthen adaptive capacity to hot weather, including improvements in heat-health education and awareness, investment in locally appropriate adaptation infrastructure, and enhanced service and transport support during extreme heat.

- Enhance heat-health education and awareness

Participants stressed the importance of adequate health awareness programs led by the local health service. Training community members to recognise signs and symptoms of heat-related illnesses, precautions and possible treatment were also suggested as a way to create awareness.

*“Go around to the community members and talk to them about the heat and how they can stay safe maybe the clinic can do that too”* [ Site 2.1].

*“The community should be trained in how to recognise heat stress in people*,* signs of heat stress and heat strokes; the clinic and health department need to come out and do more health promotion activities in the communities”* [Site 3.1].

Participants mentioned that various modes of communication, including posters, videos, documentaries, and short movies, can help communities adjust to hot weather. Suggestions included preparing awareness material in the local language.

*“Anything [video/posters] to do with the heat. And for the old people*,* there should be a video in the language so they can understand”* [Site 2.3].

*“That’s [awareness videos] the best thing to do for our people that aren’t real good at reading and writing. Once you show and tell that person*,* they will do it all the time”* [Site 1.9].

(b) Invest in local adaptation infrastructure

Participants suggested building more shaded areas and water facilities around the community parks, sports grounds, meeting grounds and around cemeteries.

*“I reckon more shades and more taps and stuff around the cemetery and the ovals and probably with the basketball courts”* [Site 1.7].

*“Shade*,* tap with the water on it or a tank like this [pointing to rainwater collection tank] to help people during hot weather”* [Site 1.4].

Climate-friendly housing that reduced reliance on energy-intensive cooling appliances was recommended across sites. Community members highlighted the need for community consultation while building houses in remote sites. Timely installation, repair and servicing of indoor cooling systems were considered extremely important.

*“…while it’s cold [cold weather]*,* they [housing department] should be servicing every air conditioner*,* in every community*,* before the summer come up and everybody’s ready to go”* [Site 4.1].

*“In these new houses here*,* it’s like sheets of iron*,* and in the hot weather*,* it gets hotter”* [Site 1.7].

Regular public transport to town during hot weather was recommended as a strategy to ensure people had access to the town’s facilities, including cool infrastructure and hospital services.

*“Sometimes it’s hot*,* you still see people walking into town just down the road. There is no transport at all. We don’t even have a public transport [public bus] service that comes out here to help people”* [Site 4.5].

*“Clinics should do the pickups*,* picking people from their houses and taking them to the clinic”* [Site 2.5].

## Discussion

Our study has highlighted that First Nations people’s adaptive capacity to climate change is complex and influenced by predominantly modifiable vulnerability factors alongside often underappreciated and undervalued resilience factors. External pressures, such as inadequate infrastructure, limited access to healthcare and socio-economic marginalisation^28^, are prevalent in remote Australia and contribute significantly to climate vulnerability. Nevertheless, this study also highlights the critical role of community strengths, including local adaptation knowledge, social support systems, and First Nations knowledge and practices in promoting heat resilience. These factors, though sometimes undervalued in mainstream climate adaptation frameworks, offer important pathways for building long-term adaptive capacity in remote First Nations communities. By understanding and addressing these dynamics, this study aims to inform more culturally appropriate and effective adaptation strategies to protect First Nations’ health and well-being in the face of a rapidly changing climate.

### Heat-health vulnerabilities in remote First Nations communities

Our findings emphasise the critical role of poor housing and socio-economic marginalisation in shaping heat vulnerability. This is consistent with other studies in which overcrowding, malfunctioning air conditioning units, and power accessibility and affordability issues have been reported as barriers to indoor cooling^8,29,30^. Overcrowding remains a longstanding issue in remote First Nations housing, with many homes accommodating multiple generations in spaces designed for far fewer occupants^29^. Overcrowded homes in summer amplify heat-related discomfort. It has been suggested that the poor thermal performance (poor insulation, lack of proper ventilation, and ineffective regulation of indoor temperatures) of existing housing and inadequate outdoor landscaping (lack of man-made shade structures, little greenery and presence of heat-reflective surfaces) force multiple inhabitants into houses^30^. This then increases risks of infectious disease transmission (acute infections can contribute to later chronic conditions), and adversely affects sleep and personal safety^31^. In addition, study participants perceived delayed repairs and maintenance of air conditioning units and malfunctioning appliances as a major concern in the remote communities, reflecting broader issues with underfunded and reactive housing policies in remote areas^32^.

Air conditioner use is a commonly reported cooling strategy for Australian urban and regional households that can afford the associated power costs^33^. However, in remote communities, energy insecurity is common and constrains access to, and sustained use of, air conditioning^8,29,34^. Electricity sources in remote communities are diverse and include diesel generators, hybrid solar-diesel systems, and in some communities, connections to the national electricity grid^8^. Many of the off-grid remote communities in the NT continue to rely on diesel generators^34^. The transition to renewable energy power sources in remote Australia is gradually progressing, with government and non-government initiatives over the past decade providing off-grid power to First Nations communities with varying degrees of access and support^29^. The provision of electricity to geographically remote communities is already a financial burden to service providers and remote households, and it is likely to worsen as climate change intensifies, bringing more frequent and extreme heat events.

In this study, community awareness of heat-related health risks varied among community members, with many participants having limited knowledge about symptoms, prevention, and appropriate responses. This knowledge gap has been reported in other remote First Nations studies^29,30^ where, for example, implementing culturally sensitive communication approaches has played a key role in strengthening resilience and mitigating heat-related vulnerabilities among diverse populations^35^. Participants’ accounts further suggest that, in the absence of accessible and culturally appropriate heat-health information, some short-term coping responses to heat, such as reliance on soft drinks or alcohol for perceived cooling, may provide immediate relief while potentially increasing health risks during hot weather. This aligns with broader discussions in the climate adaptation literature that some coping responses may have unintended consequences over time^36^, including exacerbation of dehydration, metabolic risk, or heat-related illness. Targeted heat-health education programs, co-designed with First Nations communities, are needed across remote Australia to improve awareness and reduce health risks.

As also observed by the participants in this study, research confirms that Central Australia has experienced significant reductions in surface water availability, intensifying drought conditions and reducing access to traditional water sources^37^. This not only increases heat stress and dehydration risks but also impacts cultural practices related to water use and bush resource management^38^, potentially limiting their effectiveness as a long-term adaptation measure. The decline of bush tucker and the increased dominance of buffel grass are key environmental concerns. Buffel grass expansion has been linked to both biodiversity loss and increased fire risk, further compromising food security and traditional ecological practices^39^ that affect health and well-being. The loss of traditional food will intensify heat vulnerability by reducing access to locally available hydrating sources of bush foods, forcing people to rely on processed store-bought food that often lacks adequate hydration properties and nutritional value. This will not only affect dietary intake but also weaken an important cultural adaptation mechanism.

While heat vulnerability in remote First Nations communities shares similarities with broader global patterns, including those documented among First Nations communities in North America and the Pacific Islands, there are distinct socio-environmental factors unique to Australia. Global reviews of extreme heat adaptation actions identify housing quality, energy insecurity, water access, and socio-economic marginalisation as key drivers of heat-health vulnerability across regions^40^. Indigenous-focused synthesis work further shows that although these vulnerability domains are widely shared, adaptation responses are strongly place-based and shaped by governance and access to institutional support^41^. For example, research from the Navajo Nation in the USA^42^ and First Nations communities in Canada^43^ highlights how structural inequalities, including inadequate housing and energy poverty, exacerbate heat exposure. However, these communities often have greater access to governmental support and additional support mechanisms, including philanthropic funding and collaborative adaptation initiatives, than their Australian counterparts^44^. Conversely, Pacific Island communities, facing rising temperatures, food insecurity, and water scarcity, have implemented culturally grounded climate adaptation strategies, such as water-conservatory techniques, disaster-resilient cropping, and agricultural terracing^45,46^.

### Resilience and adaptive capacity to hot weather in remote First Nations communities

Communities demonstrated immense climate resilience due to their strong local PHC services, cultural knowledge, and local adaptation practices. The PHC sector plays a critical role in addressing climate-related health impacts^47^, particularly in remote settings where mainstream healthcare access is limited. While the availability of health services during heat events enhances community resilience, challenges such as workforce shortages^48^ and infrastructure limitations, including unreliable transport access^20^ to PHC centers remain areas for policy attention. Access to community cooling centers reduces heat-related health risks in disadvantaged settings^49^. Participants acknowledged that public spaces such as schools, clinics, aged care centers, and community stores serve as cooling hubs during hot weather. These amenities were considered as potential places to connect with other community members, facilitating social networking within the communities. However, to further enhance resilience, it is crucial to address the accessibility concerns raised by participants, particularly for individuals with mobility challenges or limited transport options.

Our findings also demonstrate that remote communities in Central Australia rely on drinking plenty of water, consuming bush tucker, using creeks and waterholes for cooling, modifying daily activities, and constructing humpies (lean-tos) for shade when out hunting. These practices indicate an established understanding of the local environment and the ability to adjust behaviour to mitigate heat stress, supporting evidence that TEK is fundamental to climate adaptation^50,51^. Communities minimise heat exposure by shifting activities to early mornings or evenings while maintaining important cultural practices. Research from the East Kimberley region in Australia has similarly documented the use of the *Miriwoong* seasonal calendar to monitor small-scale climatic changes and is seen by *Miriwoong* elders as a way to adapt their resource use and land management practices to the changing environmental conditions^51^. Many of these practices function as short-term coping responses to heat stress, enabling people to manage day-to-day exposure rather than constituting long-term structural adaptation.

While western adaptation models often focus on infrastructural solutions, such as air conditioning and built environment modifications, First Nations strategies also prioritise behavioural adjustments, water-based cooling, and ecological interactions as culturally appropriate and accessible heat mitigation strategies. These were consistently identified across our study communities. Elsewhere in Northern Australia^29,30^, community members similarly use a range of indoor and outdoor cooling strategies and behavioural adaptations to cope with hot weather. This contrast highlights the importance of integrating TEK into formal adaptation policies, rather than relying solely on Western technological solutions. In addition, while government-led adaptation strategies often include standardised solutions, such as heat-health warning systems, First Nations communities may also use local weather knowledge, including changes in wind patterns, animal behaviour, and plant cycles, to predict and respond to extreme heat^45,46^. This localised approach is highly effective in culturally relevant contexts but is often overlooked in mainstream climate adaptation planning. Moreover, the blend of modern and First Nations knowledge reflects a hybrid approach to climate adaptation, where First Nations communities integrate TEK with contemporary forecasting systems to manage environmental risks^52^.

These behavioural and culturally grounded responses play an important role in reducing everyday heat exposure, but they also have limits under conditions of intensifying and prolonged extreme heat. Many of the strategies described depend on reliable access to water, safe and accessible cooling environments, and the capacity to modify daily routines, which may not always be feasible in remote or resource-constrained settings^53,54^. Their effectiveness may be constrained during extended heat events, particularly when night-time temperatures remain elevated, limiting opportunities for physiological recovery and increasing cumulative heat stress^55–57^. Structural conditions such as housing quality and energy insecurity can further limit the feasibility of behavioural adaptation. These constraints are often compounded by declining availability of culturally important environmental resources and mobility challenges for older people and those living with chronic illness^53,54,58^. Recognition of these constraints highlights that community-led strategies alone cannot fully offset heat-health risks in the absence of supportive infrastructure and services, reinforcing the need for complementary structural and system-level interventions.

In this context, incorporating First Nations knowledge into heat adaptation frameworks can support policies that are more responsive to community needs and more sustainable in the long term. Addressing heat-health vulnerabilities therefore requires cross-sector collaboration across housing, energy, climate, and health systems, ensuring that adaptation efforts are holistic, culturally appropriate, and community-driven.

### Strengths and limitations

This study prioritised the perspectives of remote First Nations people in Central Australia and foregrounded their lived experiences. By applying the linked vulnerability-resilience framework^26^, the research does not focus on disadvantages, rather, it draws out areas of strength to inform policy and practice. A key strength of the study also lies in the use of one-on-one yarning and the involvement of First Nations cultural navigators, which supported culturally safe engagement and enabled a deeper understanding of place-based knowledge, everyday coping practices, and culturally relevant adaptation strategies.

The study is context-specific, focusing on arid-zone remote First Nations communities in Central Australia. As such, the findings are not intended to be statistically generalisable to all First Nations communities, particularly those in different climatic, ecological, or governance contexts. In addition, qualitative design emphasises lived experience and perceived impacts rather than quantitative measurement of exposure or health outcomes. The study primarily documents short-term coping responses and adaptive practices, rather than evaluating long-term adaptation effectiveness or future adaptation pathways.

## Conclusion

This study examined heat-related vulnerabilities and responses to hot weather in remote First Nations communities of Central Australia through participants’ lived experiences, using culturally grounded qualitative methods. The study documents how hot weather shapes health, daily activities, and community life in one of Australia’s most climatically extreme regions.

Participants described a range of responses used to manage hot weather, including adjusting the timing of activities, seeking shade and water-based cooling, using household- and community-level cooling practices, and relying on PHC services and shared community infrastructure. However, the effectiveness of these responses was consistently determined by broader structural and environmental conditions, including housing quality, energy insecurity, environmental change, limited shaded outdoor spaces, and uneven access to heat-health information.

The findings indicate that many responses to hot weather in remote settings function primarily as short-term coping practices rather than long-term adaptation, particularly under conditions of intensifying and prolonged heat. Strengthening climate resilience therefore requires action beyond individual or household responses, including investment in climate-resilient housing, reliable and affordable energy access, community cooling infrastructure, and locally relevant heat-health communication. While the findings are context-specific and not intended to be statistically generalisable, they provide empirically grounded insight to inform co-designed, place-based approaches to reducing heat-related health risks in remote and climatically extreme settings.

## Supplementary Information

Below is the link to the electronic supplementary material.

Supplementary Material 1

## Data Availability

The datasets generated and/or analysed during the current study are not publicly available due to ethics approval conditions and consent provided by research participants. However, the datasets may be obtained from the corresponding author on reasonable request if ethics approval is obtained.
