# Supplementary material for: Exploring adaptive capacity to arid heat in remote First Nations communities in Central Australia
Source: Sci Rep. 2026 Feb 21;16:10111. doi: 10.1038/s41598-026-40677-2 (PMC13022389; doi:10.1038/s41598-026-40677-2)
Supplement: Supplementary file 1 — Supplementary Material 1 [file 41598_2026_40677_MOESM1_ESM.pdf]

Exploring adaptive capacity to arid heat in remote First Nations communities in Central Australia

Manoj Bhatta[a*], Gloria Baliva[a], Sophie Pascoe[b], Mohammad Radwanur Talukder[c], Vahab Baghbanian[d], Deborah Russell[a], Linda Ford[e], Alan Cass[b], John Wakerman[a], Supriya Mathew[a]

a Menzies School of Health Research, Charles Darwin University, Alice Springs, NT, Australia

b Menzies School of Health Research, Charles Darwin University, Darwin, NT, Australia

c Leukaemia Foundation, Adelaide, SA, Australia

d Central Australian Aboriginal Congress, Alice Springs, NT, Australia

e Northern Institute, Charles Darwin University, Darwin, NT, Australia

Supplementary File S1: Data collection guide: Researcher observations and one-on-one yarning sessions

Overview

This guide outlines the observational and qualitative data collection approaches used to document lived experiences of hot weather, adaptive practices, and community-identified priorities related to heat-health in remote First Nations communities in Central Australia. Data collection was undertaken using culturally appropriate, flexible, and relational methods consistent with First Nations research practice and qualitative ethnographic approaches.

1. Study participants

Community participants included adult First Nations residents from participating communities. Recruitment aimed to reflect diversity across gender, age, family roles (including parents and carers), and employment status, based on local knowledge and guidance from First Nations cultural navigators.

2. Data collection methods

2.1 Researcher observations

Researchers undertook contextual observations within each community to document everyday exposure to hot weather and the availability and use of local adaptation infrastructure. Observations were intended to provide contextual insight rather than systematic behavioural measurement.

Observations focused on:

Adaptation infrastructure, including:

- Access to public cooling spaces (e.g., air-conditioned buildings, art centres, community stores)
- Availability of swimming pools or water-based cooling options
- Access to potable drinking water
- Energy access and energy security
- Presence of solar infrastructure

Use of space within the community, including:

- How people used indoor and outdoor spaces at different times of day
- Presence or absence of shade in public and residential areas
- Changes in activity patterns during hot periods

Observations were conducted in public and communal spaces and informed interpretation of the qualitative findings.

2.2 One-on-one yarning sessions

Primary qualitative data were collected through one-on-one yarning sessions, conducted with support from a local First Nations researcher and First Nations cultural navigators.

Yarning sessions were conversational, participant-led, and guided by a flexible topic guide rather than a structured interview schedule. This approach supported culturally safe knowledge sharing and allowed participants to prioritise issues most relevant to their lived experiences.

Note: A yarning circle was conducted separately as a culturally grounded process to support interpretation and contextualization of findings. This process did not generate new primary data and is described in the main Methods section.

3. Sample size

A maximum of approximately 10 participants per community was sought, with overall recruitment aiming to capture variation across:

- Gender
- Older and younger adults
- Families with children

4. One-on-one yarning guide

Theme 1: Impacts of hot weather on health and primary health care use

Core questions

- How does hot weather affect your health?

- How does hot weather affect the health of your family members and people in your community?

Prompts

- Do you feel sick when it gets hot?
- What kinds of health problems do people experience on very hot days (e.g., headache, anger, confusion, dehydration)?
- Does heat affect children, older people, pregnant women, or people with medical conditions differently?
- How are older people looked after in the community?
- Is there a centre or place for elders?

Energy and housing

- Do you use power cards to access electricity?
- What happens when your power gets disconnected on a hot day?
- How do you keep your family cool when that happens?
- What do you do with food in the fridge?
- Do you have a working air-conditioner?
- Has it ever broken down during summer? How long did repairs take?
- Are windows and shading around the house adequate?

Alcohol and heat

- What have you noticed about how hot weather affects people when alcohol is involved?
- Do people get more upset or stressed in hot weather?

Health service use

- Do you or others go to the clinic more or less during hot weather?
- Who usually visits the clinic on hot days?
- Why do people go to the clinic when it is hot?
- Does the clinic do outreach visits or home checks during extreme heat?
- Does the clinic provide after-hours support?

Daily life

- How does heat affect your day-to-day activities?

  Prompts: hunting, bush tucker, sport, visiting family, shopping
- How does heat affect sleep?

- Are sleeping places comfortable during summer?

Theme 2: Past and present adaptation practices

Cooling practices

- How do you and your family keep cool on hot days?

  Prompts: indoor and outdoor strategies
- How do you keep cool inside the house?
- How do you keep cool outside the house?

Change over time

- When you were younger, how did you keep cool?
- Have you noticed changes in weather or heat over time?

Optional prompts for older participants

- Did you live in a house when you were younger?
- Did you have electricity, fans, or air-conditioning?
- How did people manage sickness before modern medicines?
- Did people use bush medicines or particular clothing?

Environmental change

- Have you noticed changes in land, water, or bush tucker?
- Are there things that now make it harder to keep cool?

Care for vulnerable groups

- How are children and older people looked after on hot days?
- What happens at the clinic during extreme heat?

Theme 3: Heat-health communication and information

- How do people get weather information?

  Prompts: TV, radio, social media, clinic
- Which channels do people prefer?
- Do preferences differ by age or employment?
- Do people use traditional knowledge to predict hot or cold weather?
- Do clinics provide heat warnings?

Theme 4: Community recommendations

- What would help keep people healthy on hot days?
- What could clinics do differently during hot weather?

- Would people like heat-health alerts?
- What type of information would be helpful?

  Prompts: posters, videos, artwork, language-based materials
- Should more people be trained to recognise heat stress?
- Are there enough shady areas and water points?
- Should more shade structures be built?

Supplementary Table S1: Deductive codebook for one-on-one yarning data aligned with the vulnerability–resilience framework

**Theme**	**Sub-theme (as in Results)**	**Definition**	**Includes**	**Excludes**	**Illustrative quote (verbatim)**
Heat-related vulnerabilities	Health and well-being	Perceived physical, mental, or social health impacts associated with exposure to hot weather	Heat illness, fatigue, respiratory symptoms, emotional distress, reduced daily or cultural activities	Health issues not linked to heat	"My daughter has asthma, she feels out of breath when she is in the heat." [Site 3.3]
Heat-related vulnerabilities	Infrastructure to adapt	Constraints related to housing, energy, water, and services that limit adaptation to heat	Broken air conditioners, lack of shade, power disconnections, delayed repairs	Individual behavioural coping	"I rang housing mob six months ago, still waiting for my aircon and broken window to be fixed, waiting too long." [Site 3.2]
Heat-related vulnerabilities	Infrastructure to adapt	Same as above	Distance to shade, uneven distribution of cooling infrastructure	Natural environmental features	"Too far walk over to the shade around the community." [Site 2.1]
Heat-related vulnerabilities	Infrastructure to adapt	Same as above	Lack of yard shade	Individual preference	"Maybe more trees, shade in the yard." [Site 2.4]
Heat-related vulnerabilities	Environmental changes	Observed environmental	Reduced rainfall, drying waterholes,	Seasonal change without	"Not enough rain, you won't fill up the aquifer or your

		changes affecting cooling opportunities and wellbeing	bushfires, loss of bush tucker	heat implications	spring, it will go dry.” [Site 1.1]
Heat-related vulnerabilities	Environmental changes	Same as above	Fire impacts on bush foods	General fire commentary	“Too many bushfires, got to wait to get a little bit of rain to bring bush tucker back.” [Site 2.4]
Heat-related vulnerabilities	Environmental changes	Same as above	Declining bush food availability	Store food issues	“Less bush tucker due to change in weather conditions and water availability.” [Site 1.2]
Heat-related vulnerabilities	Knowledge and awareness of heat-related health risks	Variation in awareness and understanding of heat-health risks and appropriate responses	Gaps in health promotion, misunderstandings of cooling practices	Service delivery not related to communication	“The only thing that's missing out is the people in the community who are not really into literacy and numeracy, who don’t know all this. Where you still need the health department to come and do promotions on those sorts of issues now, like blood sugar

					levels, diabetes, and hypertension." [Site 1.1]
Resilience to hot weather	Adaptation practices adopted by residents	Individual or household strategies used to reduce heat exposure	Shade use, activity shifting, water-based cooling	Institutional interventions	"Kids sit under the shady trees and play under the shade." [Site 4.3]
Resilience to hot weather	Adaptation practices adopted by residents	Same as above	Indoor shading	Structural housing design	"We put a sheet or blanket over the curtains on the window to darken the room down." [Site 2.3]
Resilience to hot weather	Adaptation practices adopted by residents	Same as above	Activity timing	Recreational preference	"You play football in the morning, nighttime play basketball right but in between the morning to lunchtime to the afternoon you got no other sports activities." [Site 1.1]
Resilience to hot weather	Adaptation practices adopted by residents	Same as above	Early-morning work	Occupational requirements	"In hot weather, I get up early to start cleaning up before it gets hot, like, do the yard and everything before it starts to get hot." [Site 4.3]

Resilience to hot weather	Adaptation practices adopted by residents	Same as above	Bush food consumption for cooling	Nutritional discussion not linked to heat	"We eat bush bananas that help cool down in the summertime." [Site 2.5]
Resilience to hot weather	Strong primary care	Health service actions supporting resilience during heat	Transport, advice, continuity of care	General clinic use	"We get information from the clinic (verbally) about weather warnings, like if it's going to be a heatwave in the next couple of days and stay out of the heat and that." [Site 3.1]
Resilience to hot weather	Public infrastructure and local support systems	Use of shared spaces and social networks to reduce heat exposure	Cooling hubs, sharing food or power	Individual coping without social element	"If we have meat, we will take it to someone else's house and store it for the night or till whenever power comes back on." [Site 2.1]
Community recommendations on improving adaptive capacity	Enhance heat-health education and awareness	Community-identified actions to improve heat-health knowledge	Training, culturally appropriate materials	Infrastructure-only suggestions	"The community should be trained in how to recognise heat stress in people, signs of heat stress and heat strokes; the clinic and health department need to come out and do more health promotion

					activities in the communities." [Site 3.1]
Community recommendations on improving adaptive capacity	Invest in local adaptation infrastructure	Recommendations for physical or service improvements to reduce heat exposure	Shade, water access, housing design, transport	Behavioural strategies	"I reckon more shades and more taps and stuff around the cemetery and the ovals and probably with the basketball courts." [Site 1.7]

Supplementary Table S2: Additional illustrative quotes supporting key analytical themes

Theme	Sub-theme	Illustrative quote	Site
Heat-related vulnerabilities	Infrastructure to adapt	"I rang housing mob six months ago, still waiting for my aircon and broken window to be fixed, waiting too long."	Site 3.2
Heat-related vulnerabilities	Infrastructure to adapt	"Too far walk over to the shade around the community."	Site 2.1
Heat-related vulnerabilities	Infrastructure to adapt	"Maybe more trees, shade in the yard."	Site 2.4
Heat-related vulnerabilities	Environmental changes	"Not enough rain, you won't fill up the aquifer or your spring, it will go dry."	Site 1.1
Heat-related vulnerabilities	Environmental changes	"Too many bushfires, got to wait to get a little bit of rain to bring bush tucker back."	Site 2.4
Heat-related vulnerabilities	Environmental changes	"Less bush tucker due to change in weather conditions and water availability."	Site 1.2
Resilience to hot weather	Adaptation practices adopted by residents	"Kids sit under the shady trees and play under the shade."	Site 4.3
Resilience to hot weather	Adaptation practices adopted by residents	"We put a sheet or blanket over the curtains on the window to darken the room down."	Site 2.3
Resilience to hot weather	Adaptation practices adopted by residents	"You play football in the morning, nighttime play basketball right but in between the morning to lunchtime to the afternoon you got no other sports activities" [Site 1.1].	Site 1.1
Resilience to hot weather	Adaptation practices adopted by residents	"In hot weather, I get up early to start cleaning up before it gets hot."	Site 4.3

Resilience to hot weather	Adaptation practices adopted by residents	“We eat bush bananas that help cool down in the summertime.”	Site 2.5
